# Using CNN and HHT to Predict Blood Pressure Level Based on Photoplethysmography and Its Derivatives

**DOI:** 10.3390/bios11040120

**Published:** 2021-04-13

**Authors:** Xiaoxiao Sun, Liang Zhou, Shendong Chang, Zhaohui Liu

**Affiliations:** 1 Xi’an Institute of Optics and Precision Mechanics of CAS, Xi’an 710119, China; sunxiaoxiao@opt.cn (X.S.); lzh@opt.ac.cn (Z.L.); 2 University of Chinese Academy of Sciences, Beijing 100049, China; 3 EAIT (Engineering, Architecture and Information Technology Department), University of Queensland, Brisbane 4072, Australia; shendong.chang@uqconnect.edu.au

**Keywords:** blood pressure, photoplethysmography, derivatives of PPG, convolutional neural network, ensemble empirical mode decomposition

## Abstract

According to the WTO, there were 1.13 billion hypertension patients worldwide in 2015. The WTO encouraged people to check the blood pressure regularly because a large amount of patients do not have any symptoms. However, traditional cuff measurement results are not enough to represent the patient′s blood pressure status over a period of time. Therefore, there is an urgent need for portable, easy to operate, continuous measurement, and low-cost blood pressure measuring devices. In this paper, we adopted the convolutional neural network (CNN), based on the Hilbert–Huang Transform (HHT) method, to predict blood pressure (BP) risk level using photoplethysmography (PPG). Considering that the PPG′s first and second derivative signals are related to atherosclerosis and vascular elasticity, we created a dataset called PPG+; the images of PPG+ carry information on PPG and its derivatives. We built three classification experiments by collecting 582 data records (the length of each record is 10 s) from the Medical Information Mart for Intensive Care (MIMIC) database: NT (normotension) vs. HT (hypertension), NT vs. PHT (prehypertension), and (NT + PHT) vs. HT; the F1 scores of the PPG + experiments using AlexNet were 98.90%, 85.80%, and 93.54%, respectively. We found that, first, the dataset established by the HHT method performed well in the BP grade prediction experiment. Second, because the Hilbert spectra of the PPG are simple and periodic, AlexNet, which has only 8 layers, got better results. More layers instead increased the cost and difficulty of training.

## 1. Introduction

Patients with chronic hypertension will experience serious consequences if it is left untreated, including a range of cardiovascular diseases affecting the heart [1]. But most patients have no obvious symptoms in the early stages of the disease, so it is important to check BP level regularly.

The traditional method of BP measurement uses a cuff-link-type BP meter. The “white coat effect” refers to patients taking it in a medical setting with even less accurate BP than when they take it at home [2,3]. Therefore, a single measurement datum is not enough to reflect the human condition. Continuous measured BP is more accurate than single measured BP [4]. In view of the shortcomings of clinical invasive continuous BP measurement, which is difficult to perform and may cause serious harm to patients, noninvasive continuous BP measurement is of great significance.

With increases in age and changes in the physical condition of human beings, the elasticity of the blood vessel wall will decrease. When the resistance of blood flowing in blood vessels increases, blood pressure will increase accordingly. This is the pathogenesis of hypertension. Currently, there are three most commonly used noninvasive continuous BP detection methods. The first is the pulse transit time (PTT) theory method, which refers to the time difference of the diffusion of pulse waves between two arteries in a cardiac cycle. When BP in the blood vessels increases dramatically, the vascular tension and speed of arterial pressure waves will increase, leading to shortened PTT [5]. However, obtaining the time difference requires both electrocardiogram (ECG) and pulse wave signals. It is also difficult to acquire the ECG. The second detection method is based on the morphological theory of PPG [4,6,7]. The most common way to obtain the PPG signal is to use a photoelectric sensor to detect changes in light transmitted or reflected by human skin vessels [8]. PPG represents the change of human blood volume and characterizes the systolic and diastolic processes of the heart, which are linked to BP. PPG′s first and second derivative signals are related to atherosclerosis and vascular elasticity, which are factors that influence BP. Luo Zhichang et al. [9] found that the tidal wave of the pulse wave signal of high blood pressure will gradually approach the main wave, finally merge with it, and even exceed the main wave. Therefore, the main wave of the PPG signal with hypertension looks more rounded and curved than that of normotension. Thus, PPG is closely related to BP. Therefore, PPG signal is increasingly applied to predict BP, blood oxygen, respiration rate, and other data [10,11,12]. The third method combines the characteristic points of the ECG and PPG signals to predict BP [13,14,15]. The reason for the rise of this method is that, although PPG signals feature points that contain information related to SBP (systolic blood pressure), it is not easy to determine the relationship between PPG and DBP (diastolic blood pressure). Therefore, using PPG alone is bound to lead to inaccurate BP predictions. Accuracy is improved by adding the ECG signal [16]. However, obtaining the ECG signal remains a challenge in research because of current technology limitations.

**Figure 1:**
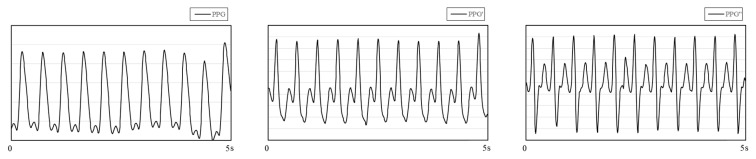
PPG (photoplethysmography) and its first derivative (PPG′) and second derivative (PPG″). The data comes from the MIMIC database.

Among the many studies on PPG signals, research on the derivatives of PPG has attracted our attention. Figure 1 shows PPG and its first and second derivatives. Qawqzeh et al. [17] analyzed the relationship between the first and second derivatives of PPG and atherosclerosis. The second derivative of PPG (SDPTG) was found to be closely related to atherosclerosis and could be used as an assistant technology to detect the disease. There are many factors causing atherosclerosis in the human body, among which hypertension is the most common one. Hypertension and arteriosclerosis cause and affect each other and exist together. Based on this, Mengyang Liu et al. [18] used PPG and its second derivatives to predict noninvasive BP. They retrained the experiment by adding 14 SDPTG features based on the 21 time-scale PPG features, and the experimental results showed that SDPTG can improve the accuracy of BP prediction. However, in real life, the morphological characteristics of PPG and SDPTG vary from person to person, and there are certain difficulties in the calibration and measurement of features, which bring great difficulty to the research. Considering the difficulty mentioned above, many scholars have innovatively used CNN to indirectly identify and extract feature points without manual calibration [19,20], which greatly reduced the time-cost. The self-adaptability of the network structure to extract feature points improves the universality of the experiment and makes the results more convincing. In addition, Liang et al. [21] found that the method using CNN has a higher accuracy than the method using PPG feature point fitting.

The purpose of our paper was to use PPG and its first and second derivative signal to predict BP level using a deep learning method. First, we segmented the PPG, extracted the baseline, and regularized the signal. Then the Hilbert–Huang Transform method was used to process the segmented signal and the generated image, and the corresponding BP value of the signal was taken as the label. Finally, the CNN was used to train the dataset. We looked at three questions; the first is to determine whether the training results of the dataset established by the HHT method are good enough on the network. The second is to explore whether more layers in the network are better for our datasets. The third is to know whether the first and second derivatives of PPG carry BP-related information.

## 2. Materials and Methods

### 2.1. Dataset Source

Both the PPG and the ABP (arterial blood pressure) data used in this paper come from the MIMIC database. Each signal has 10 s length and a sampling frequency of 125 Hz. In order to improve the generalization performance of the training frame and the generality of the experiment, in addition to the continuous and stable signal, the continuous unstable signal, and the noisy signal were retained. We divided the two signals into 2 parts for 5 s each. After signal processing—including using 0.4–8 Hz Butterworth filter as filtering, normalization, and baseline drift removal—the maximum values of systolic and diastolic blood pressure were extracted from each segment of the ABP signal, and the BP level corresponding to this segment of signal was determined by the 7th Report of the Joint National Committee on Hypertension Prevention, Detection, Evaluation and Treatment (JNC7). By analyzing the values of systolic and diastolic blood pressure (Table 1), JNC7 classifies blood pressure into three categories: normotension (NT), prehypertension (PHT), and hypertension (HT). The BP level shown by the ABP serves as the label for the corresponding segment of the PPG signal.

### 2.2. Signal Processing

PPG is a time-domain, nonlinear, and unstable human physiological signal. Therefore, processing the signal can not only retain as much information as possible contained in the original signal but can also provide images that meet the requirements for the deep learning model required by the experiment, which is the main problem we studied in this section.

Slapničar et al. [22] converted PPG and its first and second derivative signals into spectrum diagrams and input three types of spectrum diagrams into the ResNet network for training. This method required a good PPG signal, otherwise the derivative signals would become seriously deformed. Liang et al. [21] used the features of PPG to convert each segment of 5 s signal into a scalogram by using continuous wavelet transform (CWT). CWT is based on the basis function pair, which lacks adaptability and is not easily used to comprehensively describe the complex physiological characteristics of PPG, which is nonlinear and nonstationary. To make up for the shortcomings of previous studies, we decided to use the Hilbert–Huang Transform (HHT), which has the following advantages:

- The nonlinear and nonstationary problems of signals can be solved.
- The motion artifact is effectively removed from the signal.
- Spectra, after transformation, have specific physical meaning related to human physiology.
- One-dimensional signals are converted into two-dimensional signals to facilitate deep learning.

### 2.3. HHT Based on Ensemble Empirical Mode Decomposition

**Figure 2:**
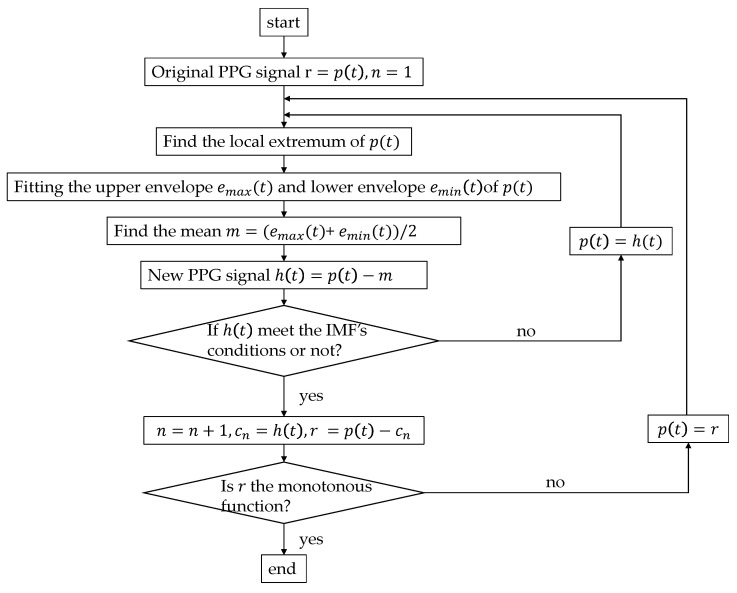
EMD (empirical mode decomposition) processing algorithm. IMF (intrinsic mode function) conditions: (1) In the entire time range, the number of local extreme points and zero points of the function must be the same or differ by one at most. (2) At any point in time, the average value of the envelope of the local maximum of the function (upper envelope) and the envelope of the local minimum (lower envelope) must be zero.

**Figure 3:**
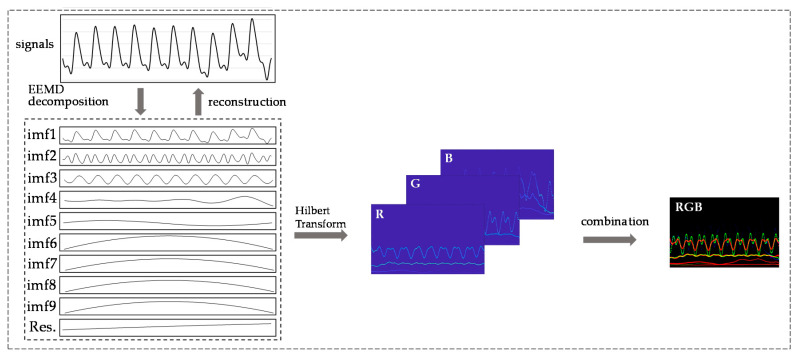
Process of processing PPG signal and its derivatives by the HHT (Hilbert–Huang transform) method.

The Hilbert–Huang Transform was proposed by N. E. Huang [23], who added the empirical mode decomposition (EMD) method on the basis of the Hilbert Transform (HT). EMD (the algorithm flow chart shown in Figure 2) can decompose nonstationary complex signals into intrinsic mode functions (IMFs). Huang et al. believed that any signal is composed of several IMFs; by filtering out the IMF components represented by the high-frequency noise signal and motion artifacts, EMD can realize the smoothing processing of nonstationary signals. By this method, the problem of morphological malformation of the PPG′s derivative signals caused by motion artifacts of PPG could be alleviated [24,25]. The IMF components of different frequencies of the decomposed PPG signal represent different physiological meanings. Previous studies have confirmed that different frequency ranges of decomposed IMFs represent different physiological activities. Mitali et al. [26] obtained surrogate respiratory signals at 0.2 Hz to 0.33 Hz. Chuang et al. [27] obtained the related information regarding heart rhythm at 0.4 Hz to 0.9 Hz. IMFs contain the local characteristics of the original signal at different time scales, so they can retain as much of the original information and characteristics of a signal as possible. Since the basis function is obtained by the EMD decomposition of the data itself, compared with the short-time Fourier transform and wavelet decomposition, the EMD method is adaptable, and the signal becomes more direct and intuitive after the Hilbert transform (Figure 3). Hilbert transformation can be performed on the decomposed IMFs to obtain spectra as inputs to the network.

However, when processing noise signals and intermittent signals, the EMD method will lead to the phenomenon of mode mixing, which seriously affects the accuracy of signal decomposition. To remedy this disadvantage, Huang et al. [28] added white Gaussian noise into the whole time-frequency space for several amounts of time, and several mean values of IMF components were obtained as the final result, which was called EEMD (ensemble empirical mode decomposition). The mode aliasing phenomenon was effectively suppressed by the EEMD method. Besides, this method could be used to remove the motion artifact of PPG signals effectively. To improve the generalization performance of the model, in addition to the serious baseline drift of some PPG signals, we selected some signals with high noise. Therefore, in this paper, we adopt the EEMD method, because this method is better than the EMD method to deal with noisy signals.

**Figure 4:**
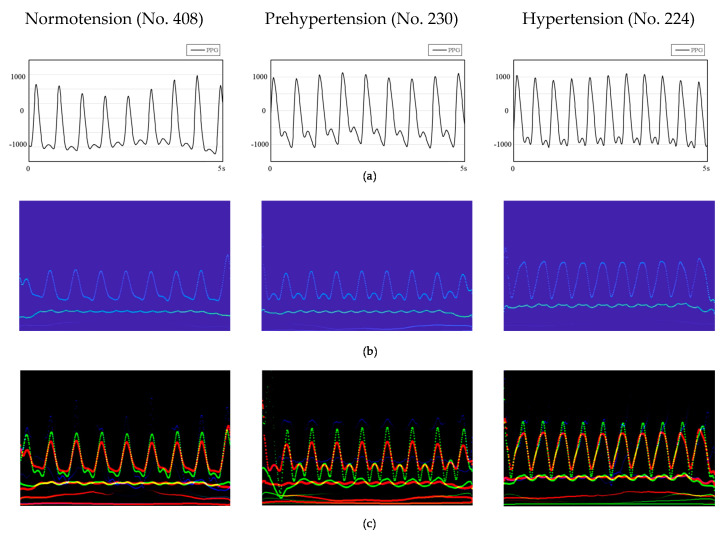
HHT spectrograms of three patients in different time periods. (**a**) PPG signals. (**b**) HHT spectra of PPG signals. (**c**) RGB images of PPG and its first and second derivative (PPG+) spectrogram combination. No. 408, No. 230 and No. 224 are the numbers of the patients.

The signal processed by the EEMD method is still a one-dimensional vector. Compared with this data format, the features of the signal shown by the two-dimensional image are more prominent. Combining the advantages of convolutional neural networks in the field of image recognition and its own characteristics, images with more prominent feature points require fewer layers, fewer calculations, and less complexity. Furthermore, the spectra of the PPG signal after HHT are related to the time-instantaneous frequency–energy distribution map. Observing the spectra of normotension and hypertension samples (Figure 4b), we found that the instantaneous frequency of the energy distribution in the spectra of the latter is higher than that of the former. Such significant information cannot be obtained using a one-dimensional vector to represent the PPG signal. Therefore, we used a two-dimensional signal as the input of the convolutional neural network.

We built the PPG+ dataset [29]; the PPG′s Hilbert spectrogram is the red channel of the RGB image. The spectrograms of the PPG′s first and second derivatives are the other two channels (Figure 3) to improve the information dimension and enhance the image′s extractable features. These three channels combine to form an RGB image with resolution of 1247 × 770 (Figure 4c); the image length is 5 s (0–5 s), and the width is 13 Hz (0–13 Hz). The sampling quality of the images is 24 bytes, which is 8 bits for each channel and 256 levels. As the input to the network, HT spectra require preprocessing such as random aspect ratio cropping, random flipping, and center cropping before entering the network. This approach is important to enhance the data. Then, we normalized the processed image, and the image size became 3 × 224 × 224. Furthermore, the control experiment’s inputs were only the PPG signals′ spectrograms.

### 2.4. Model Fine-Tuning

Transfer learning is a branch of machine learning. The aim is to deal with a new problem by transferring it to a problem that has been resolved. Transfer learning can greatly reduce the experimental costs and training time of deep learning models and also can be applied to the problem of small datasets. Many studies have shown that transfer learning can improve the generalization ability of models [30].

Fine-tuning is a widespread practice in transfer learning, and it is suitable for small datasets. One common practice is to remove the last layer of the pretrained network and replace it with a new softmax layer, which relates to a specific problem. Considering the characteristics of the model′s inputs, we used the AlexNet [31], ResNet18 [32], ResNet34 and GoogLeNet to train the data. AlexNet has five layers of convolutional layers and three layers of fully connected layers. The ReLU, as the activation function, solves the gradient dispersion problem. ResNet adds a residual block through a short-circuit mechanism between every two layers of the ordinary network, transforms the difficult identity mapping problem into a residual problem, solves network performance degradation with increasing depth, and effectively alleviates the gradient dispersion or gradient explosion phenomenon of deep neural networks. GoogLeNet has 22 layers. It connects multiple inception blocks in series with other layers to improve the expressive ability of the network. Compared with other networks, GoogLeNet has fewer training parameters and faster convergence speed. The input size of GoogLeNet is 3*299*299, which is different than the other three networks.

**Figure 5:**
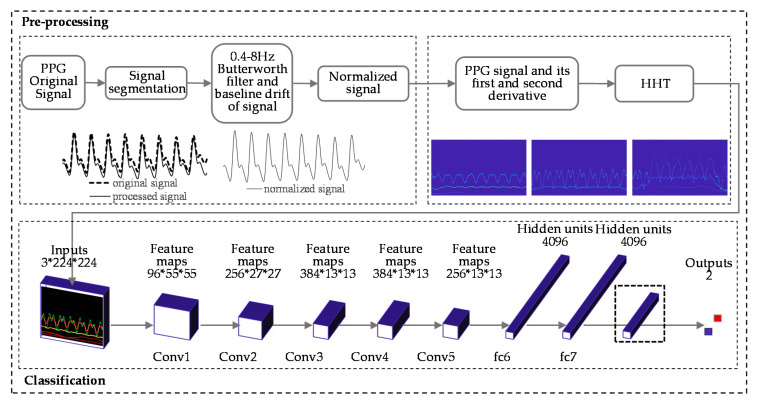
PPG signals processing procedure and the classification experiment (AlexNet).

In our work, the fine-tuning method removed the last fully connected layer when training with AlexNet, added Dropout to ignore some neurons to prevent the model from overfitting randomly, and finally added a fully connected layer with two outputs. When it came to ResNet18 and ResNet34, the specific method was to reinitialize the last fully connected layer and a linear layer with 512 input features and two output features. The parameters did not need to be trained from scratch but only fine-tuned based on the original model parameters, which dramatically improved the training efficiency and saved on training costs. Figure 5 shows the preprocessing of the signal and the classification experiment.

### 2.5. Classification Experiment

We built three classification experiments by collecting 582 data records from the MIMIC database: HT (hypertension) vs. NT (normotension), HT vs. PHT (prehypertension), and (HT + PHT) vs. NT. Two datasets, PPG and PPG+, were trained on the following networks with different layers: AlexNet, ResNet18, GoogLeNet, and ResNet34. Three two-classification experiments were performed on each dataset for every network. We designed the experiment for three purposes: First, to explore whether using our dataset to predict blood pressure levels could get better results. Second, to verify whether the PPG+ dataset containing the information of the PPG itself and its first and second derivatives was better than the PPG dataset for blood pressure prediction. Third, to consider whether the higher the number of layers, the better the prediction results would be.

The programming language used for the experiment was Python; the library used to call the model structure was PyTorch, and the model was trained on Anaconda. Code was run using a desktop with an Intel i5-8500 as the CPU, 8GB RAM, and AMD Raden R5 430 as the graphic card. After testing and optimizing the model many times, we chose Adam as the optimizer of the model, and the learning rate parameter of the optimizer was set to 0.001. Since our dataset was relatively small, we divided all data into training sets and test sets in to the ratio 7:3. To improve the generalization performance of the model and get more accurate results, we applied k-fold cross-validation to train the dataset and averaged the results. Taking into account the limitations of computer performance and the time-cost required for cross-validation, the value of k here was 5. We performed 25 epochs for each fold. The fine-tuned AlexNet used a total of 57.01M parameters, and the amount of calculation (floating point operations) was 0.71 GMAC (Memory Access Cost). After testing, the model worked best when the dropout value was set to 0.6 (for AlexNet).

## 3. Results

In this paper, we randomly selected 582 data records of ABP and PPG from the MIMIC database; each record was 10 s in length. We divided the 2 signals into 2 segments of 5 s each. Then the signal was filtered with a 0.4–8 Hz Butterworth filter and normalized. The EEMD method was used to remove the signal′s motion artifacts and baseline drift. According to the blood pressure classification standard from JNC7, the ABP of each segment was analyzed and then classified as the label of the corresponding PPG signal. Then we performed Hilbert processing on the PPG and made two datasets of PPG and PPG+. The images of the PPG+ dataset contain the first and second derivatives of PPG in addition to the signal itself. Finally, we put these two datasets into four different layers of network models for training. The results (Table 2) are represented by the F1 score, TPR (true positive rate), and TNR (true negative rate).

We reached the following conclusions from the table:

- Our dataset performed well on convolutional neural networks, especially AlexNet. In the NT vs. HT experiment, the F1 score was close to 99%, followed by (NT + PHT) vs. HT, and the training effect was the worst in the NT vs. PHT. The two datasets showed similar results on all the mentioned CNNs.
- When it came to the same network on the different datasets, the CNN performed better on the PPG+ dataset than on the PPG dataset. The improvement of the F1 score was evident in the NT vs. HT experiment of AlexNet.
- Compared to the results of different CNNs on the same experiment on the PPG+ dataset, we found that, although AlexNet has the least number of layers, the F1 score was the highest on the experiment of NT vs. HT and (NT + PHT) vs. HT. As the number of convolutional layers increased, the result did not improve but did decrease. For NT vs. HT, the effect of ResNet34 of 34 layers was improved compared with ResNet18 of 18 layers, but only a little.

From the results, the F1 score of AlexNet for our dataset was high: 98.90%. In the NT vs. HT experiment of AlexNet by using the PPG+ dataset as an example, TPR refers to the sensitivity of the model, which means that the number of normal blood pressure samples predicted by the model accounting for 99.27% of the actual normal blood pressure samples. TNR refers to the specificity of the model, which presents the sample size of hypertension predicted by the model to be 98.31% of the actual sample size of hypertension.

**Figure 6:**
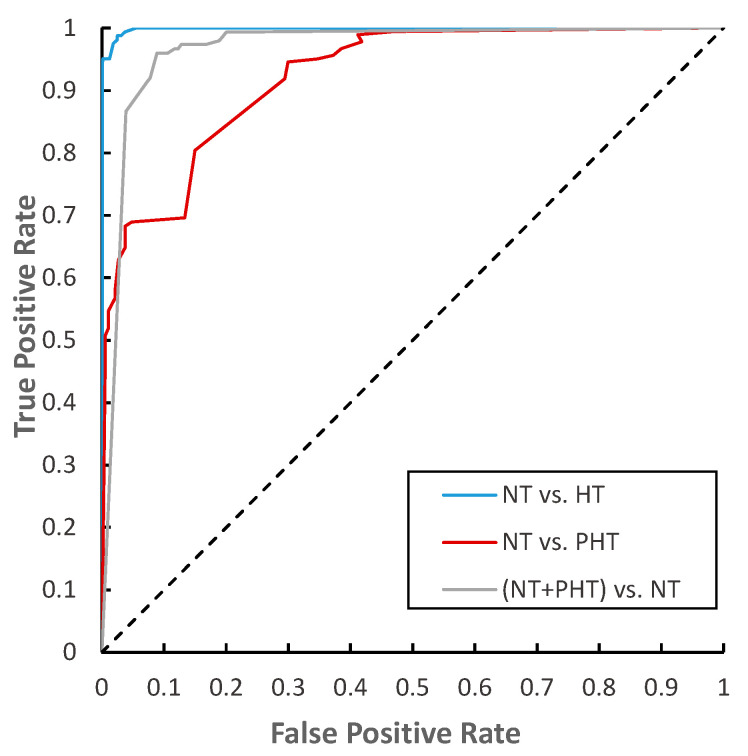
The ROC (receiver operating characteristic) curve of the three classification trials of AlexNet.

The ROC (receiver operating characteristic) curve (Figure 6) characterizes the generalization ability of the learner from the perspective of threshold selection. The closer it is to the upper left corner, the lower the classification error rate of the model, the better the threshold at this time, and the fewer false positives and false negatives there are. We found that the ROC curve of the experiment of NT vs. HT is closest to the upper left corner, which means our network performed best on it.

**Figure 7:**
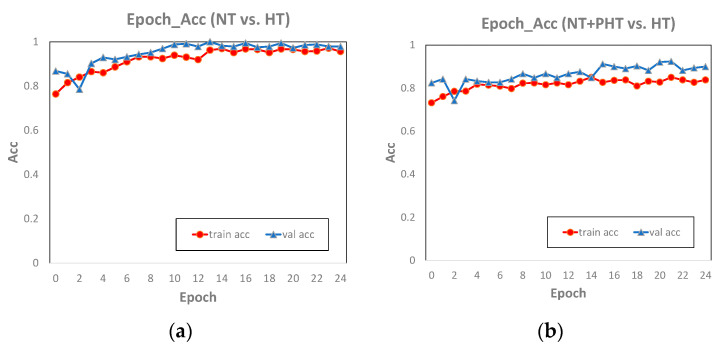

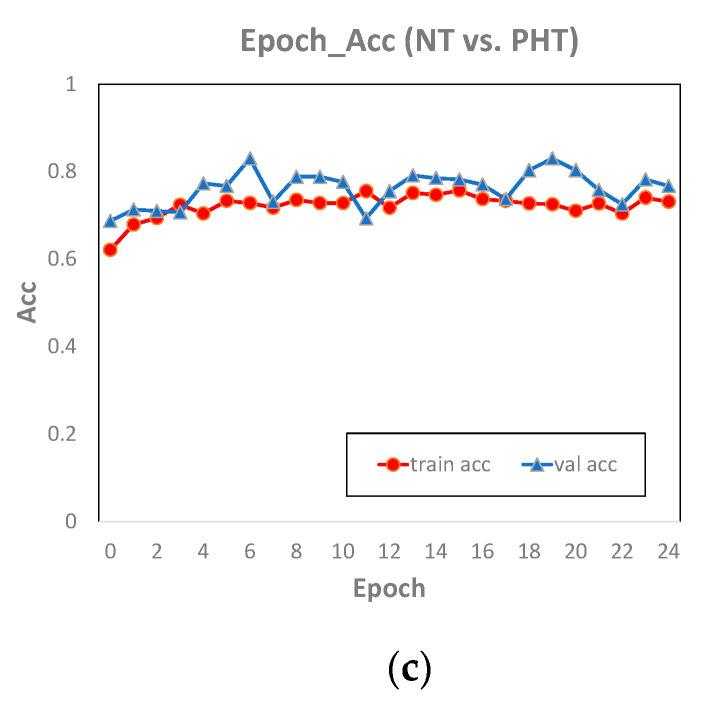
The diagram of epoch and accuracy (referred to as acc in the figure) of the NT vs. HT (**a**), NT + PHT vs. HT (**b**) and NT vs. PHT (**c**) in AlexNet.

Comparing the classification accuracy of AlexNet on the training set and the test set (Figure 7), we found the epoch_accuracy curve of the training set and the test set were very close, which means that the model performed well on our dataset, and there was neither overfitting nor underfitting. The accuracy curves of NT vs. HT and (NT + PHT) vs. HT were close to stable, indicating that the two have converged and the number of training epochs was sufficient. The accuracy of NT vs. PHT on the test set fluctuates greatly, and it has not converged yet, indicating that 25 epochs is not enough for this experiment. However, through the performance on the training set, we can basically infer the future trend of the accuracy curve of the test set. Therefore, upgrading hardware equipment and increasing the number of epochs in the experiment to improve the training effect is part of our future work.

## 4. Discussion

We used a convolutional neural network to predict blood pressure levels using PPG and its first and second derivative signals. The HHT method was applied to remove the baseline and motion artifact of PPG signals, and then we converted the one-dimensional signal into spectra as the input of the network. The blood pressure level represented by ABP was used as the label of each spectrum. According to the information contained in spectra, we established two datasets. One was called PPG+, which contained PPG itself and its first and second derivative signals. The other one was called PPG, which only contained the information in the PPG signal itself. Three binary classification experiments for each dataset were trained on four network models with different layers, AlexNet, ResNet18, GoogLeNet, and ResNet34. This study found that, although AlexNet had a small number of layers, the training results were still the best on both datasets. The fine-tuned AlexNet performed well in the NT vs. HT experiment, with an F1 score of 98.90%. The result of experiments confirmed that the first and second derivatives of PPG could improve training accuracy.

We noticed that the model performed best in NT vs. HT and performed poorly in NT vs. PHT. ROC showed that NT vs. HT was closest to the upper left corner; the distributions of these two types of data were far apart and easier to separate. The model performed poorly in the HT vs. PHT experiment because the two types of data distribution were much closer. In comparison, we also found that the PPG+ dataset had better performance than the PPG dataset in the BP classification model, especially in the NT vs. HT experiment. This confirmed that the first and second derivatives of the PPG signals carry information about BP.

The first derivative of the PPG signal represented the blood flow velocity in the aorta, and the second derivative signal represented the change in blood flow velocity (Figure 4c), which is decided by the blood viscosity and elasticity of the blood vessel wall. The hypertensive patients′ blood pressure is high, so when the aortic valve opens, blood flows into the aorta quicker. If the blood vessel elasticity is lacking, the PPG signal′s descending branch will be steeper than in ordinary people. The second derivative of the PPG signal happens to reflect this. This is the reason that adding PPG derivative information to the dataset can improve the accuracy of BP prediction.

In order to improve on the weak points of previous studies (Table 3), this paper adopted the EEMD method to process signals. The resulting HHT spectra contained the physical meaning of a particular PPG signal. The PPG signal represents the change of blood volume in human blood vessels, and the derivatives of PPG signal contain information related to blood flow. Therefore, we added the first and second derivative signals of PPG signals to the model to train on simultaneously.

The purpose of converting a one-dimensional signal into a spectrum was not only to satisfy the input format of the network model but also to make the information contained in the PPG signal more prominent. The experimental results showed that the smaller the number of layers, the better the training effect on our dataset. This result proved our approach: the feature points become more obvious after the PPG one-dimensional signal is converted into spectra, and better results can be learned without too many layers of convolutional neural networks. This can reduce the cost of model training. Compared with image recognition and classification problems such as cats and dogs, the input images in this paper had fewer feature points and lower complexity. This might be one of the reasons that the accuracy decreased or remained unchanged with more network layers in the experimental results.

Since the dataset mentioned in the paper [21,22] was not available, we used GoogLeNet to train the dataset for comparison. The F1 score of NT vs. HT in the PPG+ dataset was 89.24%. Although the result was poor compared with previous research, this result could not be evaluated arbitrarily because of the different datasets. What was certain, however, was that this result again confirmed our point that our dataset performed better on a network with fewer layers. AlexNet with only eight layers performed better on both datasets than some other networks with more layers. On the contrary, GoogLeNet, which has many layers and many complex inception blocks, did not perform well on our datasets.

## 5. Conclusions

In this paper, we used the HHT method to establish a new dataset. The fine-tuned AlexNet performed well on our dataset. The F1 score of the NT vs. HT binary classification experiment can reach 98.90%. The signals processed by HHT have specific physical meanings and obvious feature points, which were conducive to the learning of neural networks. By comparing the performance of blood pressure classification experiments in different network models, our study proved that the derivatives of PPG carry important information on blood pressure, which means that PPG and its derivatives can be used to replace the combination of ECG and PPG for blood pressure prediction. We also found that a network structure with fewer layers had a better performance on our dataset. This can reduce the amount of calculation and the time-cost of network training. We combined the EEMD method with deep learning, providing new ideas for modern medical health testing while providing a noninvasive, fast, and low-cost BP level assessment method for families and low- and middle-income countries. However, this technology still has lots of room for improvement. Our next target will focus on how to improve the classification accuracy and how to predict BP values through deep learning and will explore more information related to physiological activities from PPG and its derivative signals.

## Figures and Tables

**Table 1:** Blood pressure classification according to JNC7.

Classification	Systolic Blood Pressure (mmHg)		Diastolic Blood Pressure (mmHg)
normotension	<120	and	<80
prehypertension	120–139	or	80–89
hypertension	>140	or	>90

**Table 2:** Classification performance of the proposed deep learning method. TPR stands for true positive rate and refers to the sensitivity of the model. TNR is short for true negative rate and refers to the specificity of the model.

CNN	Layers	Trail	PPG			PPG+		
			F1 Score	TPR	TNR	F1 Score	TPR	TNR
AlexNet	8	NT vs. HT	96.33%	94.69%	97.93%	98.90%	99.27%	98.31%
		NT vs. PHT	80.35%	78.98%	84.08%	85.80%	95.26%	71.88%
		(NT + PHT) vs. HT	90.79%	90.39%	91.36%	93.54%	95.32%	91.74%
ResNet18	18	NT vs. HT	93.94%	94.51%	93.17%	94.09%	95.36%	92.33%
		NT vs. PHT	82.34%	82.85%	82.89%	84.37%	84.51%	84.59%
		(NT + PHT) vs. HT	87.35%	90.06%	84.62%	88.52%	88.87%	88.92%
GoogLeNet	22	NT vs. HT	89.48%	88.79%	90.61%	89.24%	90.19%	88.26%
		NT vs. PHT	78.05%	77.73%	79.95%	80.03%	80.79%	77.47%
		(NT + PHT) vs. HT	84.04%	81.51%	88.30%	83.46%	83.76%	83.14%
ResNet34	34	NT vs. HT	93.04%	93.04%	93.41%	94.01%	93.85%	94.26%
		NT vs. PHT	81.33%	81.75%	82.75%	84.77%	83.71%	86.34%
		(NT + PHT) vs. HT	86.76%	88.00%	83.23%	88.39%	87.15%	90.19%

**Table 3:** Comparison with well-established related work in terms of data source, feature, signal processing, and method.

Author	Data Source	Feature	Signal Process	Method
Slapničar et al. [22]	MIMIC (510 subjects over 700 h)	PPG, PPG′, PPG″	Spectro-temporal	ResNet
Liang et al. [21]	MIMIC (121 data records for 120 s each)	PPG	CWT	GoogLeNet
Our work	MIMIC (582 data records for 10 s each)	PPG, PPG′, PPG″	EEMD	AlexNet

## Data Availability

The signal data in the MIMIC database were downloaded from the following website: https://archive.physionet.org/cgi-bin/atm/ATM. The dataset we created is available at the following link: http://figshare.com/articles/figure/Blood_pressure_classification_experiment/13498422/1.
